# Long-Term Exposure to PM_2.5_ and Mortality: A Cohort Study in China

**DOI:** 10.3390/toxics11090727

**Published:** 2023-08-24

**Authors:** Jingjing Hu, Luhua Yu, Zongming Yang, Jie Qiu, Jing Li, Peng Shen, Hongbo Lin, Liming Shui, Mengling Tang, Mingjuan Jin, Kun Chen, Jianbing Wang

**Affiliations:** 1 Department of Public Health, and Department of Endocrinology of the Children’s Hospital, Zhejiang University School of Medicine, National Clinical Research Center for Children’s Health, Hangzhou 310058, China; 2 West China School of Public Health and West China Fourth Hospital, Sichuan University, Chengdu 610065, China; 3 Department of Chronic Disease and Health Promotion, Yinzhou District Center for Disease Control and Prevention, Ningbo 315040, China; 4 Yinzhou District Health Bureau of Ningbo, Ningbo 315040, China; 5 Department of Public Health, Fourth Affiliated Hospital, Zhejiang University School of Medicine, Hangzhou 310058, China; 6 Department of Public Health, Second Affiliated Hospital, Zhejiang University School of Medicine, Hangzhou 310058, China

**Keywords:** fine particulate matter, long-term exposure, mortality, prospective cohort study

## Abstract

We investigated the association of long-term exposure to atmospheric PM_2.5_ with non-accidental and cause-specific mortality in Yinzhou, China. From July 2015 to January 2018, a total of 29,564 individuals aged ≥ 40 years in Yinzhou were recruited for a prospective cohort study. We used the Cox proportional-hazards model to analyze the relationship of the 2-year average concentration of PM_2.5_ prior to the baseline with non-accidental and cause-specific mortality. The median PM_2.5_ concentration was 36.51 μg/m^3^ (range: 25.57–45.40 μg/m^3^). In model 4, the hazard ratios per 10 μg/m^3^ increment in PM_2.5_ were 1.25 (95%CI: 1.04–1.50) for non-accidental mortality and 1.38 (95%CI:1.02–1.86) for cardiovascular disease mortality. We observed no associations between PM_2.5_ and deaths from respiratory disease or cancer. In the subgroup analysis, interactions were observed between PM_2.5_ and age, as well as preventive measures on hazy days. The observed association between long-term exposure to atmospheric PM_2.5_ at a relatively moderate concentration and the risk of non-accidental and cardiovascular disease mortality among middle-aged and elderly Chinese adults could provide evidence for government decision-makers to revise environmental policies towards a more stringent standard.

## 1. Introduction

Fine particulate matter (PM_2.5_), airborne particulates with an aerodynamic diameter ≤2.5 μm [1], attracts extensive attention worldwide due to its serious pollution situation and detrimental health effects [2]. The Global Burden of Disease (GBD) study reported that the average PM_2.5_ concentration in 204 nations and regions was 42.6 μg/m^3^ in 2019 [3], whereas PM_2.5_ levels were significantly higher in developing countries [4,5]. Nowadays, epidemiologic findings have indicated that long-term exposure to ambient PM_2.5_ raises the onset and progression of respiratory and cardio-cerebral vascular diseases, such as respiratory impairment, chronic obstructive pulmonary disease, asthma, lung cancer, atherosclerosis, heart failure, and stroke [6,7,8,9,10]. Some studies suggest that exposure to PM_2.5_ may also be associated with increased risk of diabetes mellitus, osteoporosis, immune-mediated diseases, and adverse birth outcomes [11,12,13,14,15,16,17,18,19]. However, the majority of the epidemiological information was found in Europe and North America, and limited evidence is available from developing countries.

China is one of the developing countries with a significant issue of PM_2.5_ pollution [2,4] and contributes to 1425.2 thousand deaths and a corresponding huge premature death economic loss annually, owing to long-term exposure to PM_2.5_ levels beyond the WHO air-quality guidelines [2,20]. Although some previous cohort studies in China indicated that long-term exposure to PM_2.5_ was associated with excess all-cause or non-accidental mortality [21,22,23,24], the strength of association estimated in previous studies varied from each other, and the results of PM_2.5_-related cause-specific death risks were still inconsistent [21,24,25,26].

Herein, the purpose of this study was to evaluate the relationship of long-term exposure to PM_2.5_ with non-accidental and cause-specific mortality and potential interactions between PM_2.5_ and demographic and lifestyle factors.

## 2. Materials and Methods

### 2.1. Study Sample and Design

**Figure 1:**
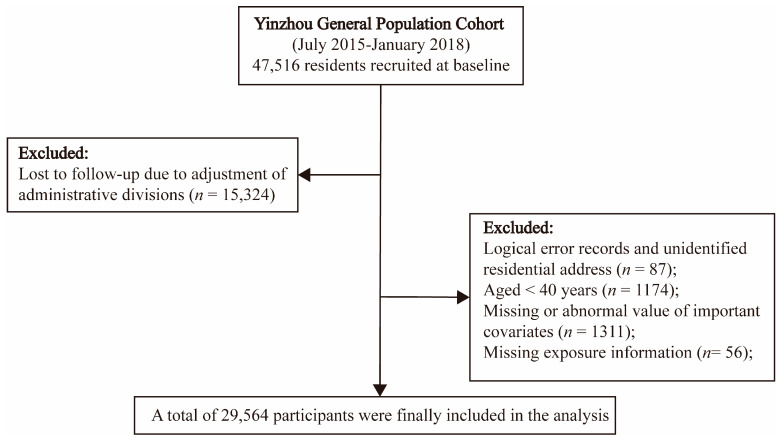
Flow chart of inclusion and exclusion of the participants in the current study.

From July 2015 to January 2018, a total of 47,516 individuals from ten towns in Yinzhou district of Ningbo, China, were selected for interviews and physical examination. We excluded 15,324 individuals lost to follow-up due to adjustment of administrative divisions. Individuals aged ≥40 years with complete data on covariates, addresses, and exposures voluntarily enrolled in this cohort (Figure 1). Each participant was followed up to death or 30 September 2021, whichever came first. The Institutional Review Board of Zhejiang University School of Medicine ethically approved our study. All participants offered informed consent at the commencement of the study.

Baseline information, including demographic factors (age, gender, occupation, educational level, marriage status, household income, height, and weight), lifestyles (alcohol drinking, smoking status, tea drinking, sleep quality, and preventive measures on hazy days), residential addresses, and chronic disease history, was inquired about by well-trained medical staff. Body mass index (BMI) was determined by dividing kilogram weight by their height in meters squared. Smoking status was divided into 2 groups (“never” and “current/former” smokers who had smoked more than one cigarette daily for over one year). Alcohol drinking was reported as “never” and “current/former drinkers”, which was characterized as consuming more than 100 g of alcohol per week within the last year. Similarly, tea drinking was classified as “never” and “current/former drinkers”, who drank tea at least twice a week over two months. A history of chronic diseases was defined as any diagnosis of chronic diseases, including hypertension, diabetes, coronary disease, asthma, chronic bronchitis, and malignancy.

### 2.2. Exposure Measures

A land-use regression (LUR) model was performed to predict the monthly concentrations of PM_2.5_. Particulars can be accessed elsewhere [27]. In a nutshell, data on PM_2.5_ and meteorological monitoring from January 2013 to December 2017 were extracted from monitoring stations with technical assistance from the Qingyue environmental protection information technology service center (http://data.epmap.org/) and the Chinese Ecology and Environment Ministry. The spatial–temporal fluctuation in PM_2.5_ was interpreted by the generalized additive model (GAM). The proximity to roads, land cover (percentage of land cover in different buffers), latitude, longitude, altitude, population density, and meteorological data were all considered as potential land-use predictors. We utilized 10-fold cross-validation to evaluate the LUR model, and the R^2^ value was 0.75 (for more details, see the Supplementary Materials). Then, we converted each participant’s residential address into a standard format recognized by the Geographic Information System (GIS) and determined exposure by averaging PM_2.5_ concentrations from 2 years before enrollment for each participant.

### 2.3. Case Ascertainment

Data on mortality were obtained from death registration system through a reliable information system (Yinzhou Health Information System, YHIS), including individual ID, death date, and cause of death, which were categorized by the International Classification of Diseases, 10th Revision (ICD-10). In our study, we focused on non-accidental deaths (A00-R99), respiratory deaths (J00-J99, C33, C34, C39), cancer deaths (C00-D48), lung cancer deaths (C33, C34, C39), and cardiovascular deaths (I00-I99).

### 2.4. Statistical Analysis

We described continuous and categorical variables as median (interquartile range, IQR) and count (percentage), respectively. Accordingly, differences between groups were tested by the Kruskal–Wallis test and the chi-squared test.

We utilized a Cox proportional-hazards model to calculate hazard ratios (HRs) and 95% confidence intervals (CIs), which were assessed for each 10 μg/m^3^ increment of PM_2.5_. We considered four models with different covariates. Covariates considered in model 1 were age and gender. Model 2 further adjusted for educational level, marriage status, occupation, and household income. In model 3, we added lifestyles, including smoking status, alcohol drinking, tea drinking, sleep quality, preventive measures on hazy days, and BMI. Additionally, model 4 adjusted for comorbidities (yes/no) based on model 3. Tests for trends in quantiles were examined using the median of quantile for exposure as a continuous variable. In order to assess the potential nonlinear concentration–response association [22,28], we fitted the concentration–response function with restricted cubic splines (RCS). The degree of freedom (df) for RCS was selected via the Akaike information criterion (AIC) [29], and the optimum df was 3 with the minimum AIC (Supplementary Table S2).

We also conducted stratified analysis to investigate effect modification by the following variables: age at enrollment (<65 years vs. ≥65 years); sex (male vs. female); educational level (illiteracy vs. literacy); occupation (industry or agriculture vs. other); household income (<30,000 CNY vs. ≥30,000 CNY); BMI (18.5–24 kg/m^2^ vs. <18.5 kg/m^2^ vs. ≥24 kg/m^2^); smoking status (never vs. current/former); alcohol drinking (never vs. current/former); tea drinking (never vs. current/former); sleep quality (good vs. bad); preventive measures on hazy days (yes vs. no); and comorbidities (yes vs. no). Multiplicative interaction terms added in model 4 were used to examine the potential interactions.

In order to test whether the results were robust, we performed six sensitivity analyses as follows: (1) excluding subjects who died during the first year after enrollment; (2) averaging PM_2.5_ concentrations from 1 year before the baseline as the exposure level; (3) excluding participants with major chronic diseases at baseline (stroke, malignant tumor, and liver cirrhosis); (4) including participants with missing covariates and using multiple imputations; (5) using a competing risk model; and (6) using a propensity-score-weighting method to control the influence of potential confounders.

All tests adopted a 0.05 two-tailed *p*-value as their significance threshold. We conducted the analyses in R version 4.1.2.

## 3. Results

In this study, we included 29,564 participants. As indicated in Table 1, the median age at enrollment was 63.17 years old (IQR: 57.04–69.28), and nearly half of the participants were male (41.1%). During the follow-up period (4.88 years, IQR: 4.14–5.15), a total of 1171 participants died: 1082 died from non-accidental events, 215 died from respiratory disease, 451 died from cancer, 125 died from lung cancer, and 409 died from cardiovascular disease.

**Figure 2:**
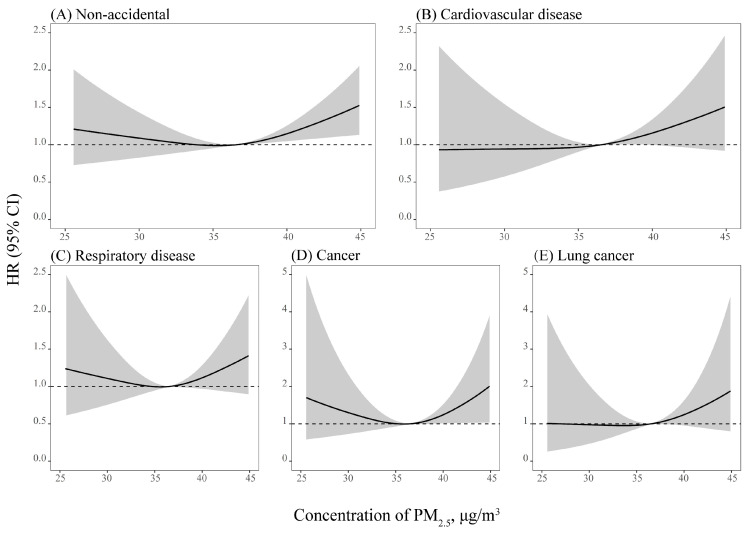
Concentration–response curves and 95% confidence intervals (CIs) for the association between a 10 μg/m^3^ increment in 2-year average PM_2.5_ and mortality based on the model 4.

The median of the average 2-year PM_2.5_ was 36.51 μg/m^3^ (range: 25.57–45.40 μg/m^3^) (Supplementary Table S1). Table 2 displays the main results of the association of each 10 μg/m^3^ increment in PM_2.5_ with mortality in different models. The HRs (95% CI) were 1.25 (1.04–1.50) for non-accidental mortality and 1.38 (1.02–1.86) for cardiovascular disease mortality after adjusting for potential covariates. Compared with the reference group, subjects in Q4 (the highest quartile) had a higher risk of non-accidental death but not for cardiovascular disease mortality. For cancer, lung cancer, and respiratory disease mortality, risk estimates were over one, but they were not statistically significant. Figure 2 shows the concentration–response curves using splines, with the optimum *df* (*df* = 3) selected via the AIC. The non-accidental and cardiovascular disease mortality were relatively flat, and then increased rapidly at the cutoff of 36.5 μg/m^3^ of PM_2.5_, but the overall associations of long-term exposure to PM_2.5_ with non-accidental and cardiovascular disease mortality were linear across the spectrum of concentrations in this cohort. In addition, the potential nonlinear relationships between PM_2.5_ and cancer, lung cancer, and respiratory disease mortality were not statistically significant either.

**Figure 3:**
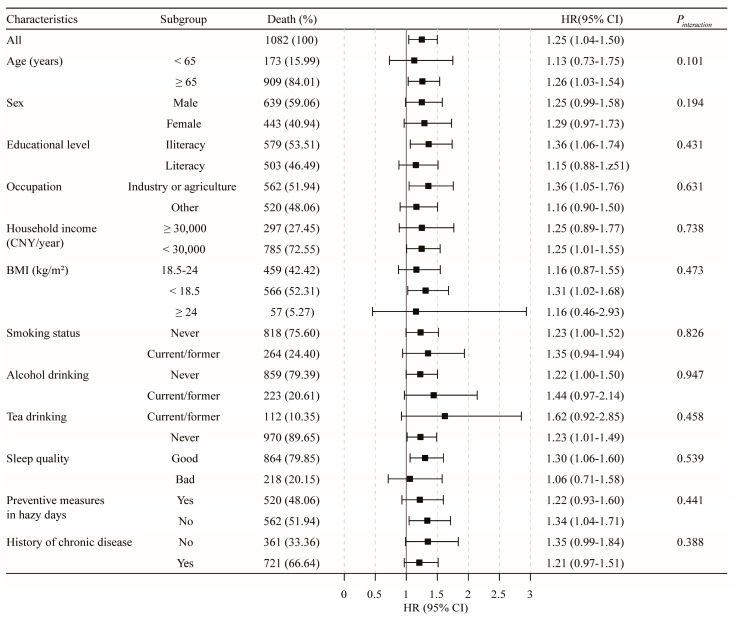
Subgroup analysis for the association of PM_2.5_ in 10 μg/m^3^ increments with non-accidental mortality.

**Figure 4:**
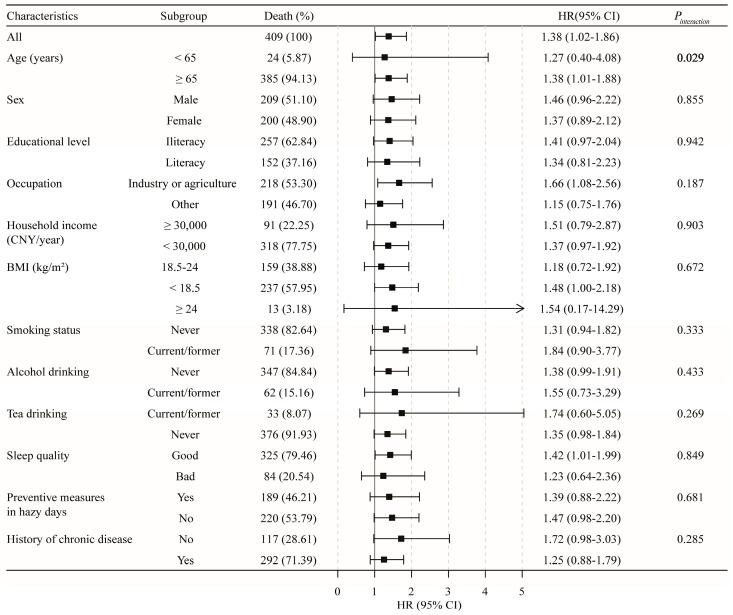
Subgroup analysis for the association of PM_2.5_ in 10 μg/m^3^ increments with cardiovascular disease mortality.

In the stratified analysis (Figure 3), the results for the relationship of PM_2.5_ with non-accidental mortality did not materially change across the subgroups by age, gender, educational level, household income, occupation, BMI, alcohol drinking, smoking status, tea drinking, sleep quality, preventive measures on hazy days, or comorbidities. Similar results were also observed in cancer and lung cancer mortality (Figures S1 and S2). However, interaction effects were observed for PM_2.5_ and age in cardiovascular disease mortality (*p* for interaction = 0.029, Figure 4) and for PM_2.5_ and preventive measures on hazy days in respiratory disease mortality (*p* for interaction = 0.040, Figure S3). Specifically, the association between PM_2.5_ and cardiovascular disease mortality was stronger among subjects aged ≥65 years (HR (95% CI): 1.38 (1.01–1.88) vs. 1.27 (0.40–4.08)), and a similar stronger association between PM_2.5_ and respiratory disease mortality was observed among individuals taking no preventive measures on hazy days (HR (95% CI): 1.78 (1.03–3.07) vs. 1.02 (0.56–1.83)).

Sensitivity analyses showed our results were not materially altered by excluding subjects who died during the first year of enrollment or those with major diseases at baseline, utilizing the 1-year average concentrations before enrollment as PM_2.5_ exposure, using multiple imputation for missing data, and using a competing risk model or a propensity-score-weighting method (Figure S4).

## 4. Discussion

In this study, we found that long-term exposure (previous 2 years) to PM_2.5_ elevated non-accidental and cardiovascular disease mortality among individuals aged ≥40 years after adjusting for potential confounders. Stratified analyses indicated stronger associations in older participants for cardiovascular disease mortality and in participants who did not take any preventive measures on hazy days for respiratory disease mortality.

To date, longitudinal studies have revealed that long-term exposure to PM_2.5_ raises non-accidental mortality to some extent [21,24,25,26,30,31]. In line with foregone cohort studies, we found a positive relationship between PM_2.5_ and non-accidental mortality, but the estimated HR was much higher in our study than that in European and North American cohorts and some Chinese cohorts, even though the concentrations of PM_2.5_ were entirely different [21,25,31,32]. For each 10 μg/m^3^ increment in PM_2.5_, pooled analysis of cohorts in China [24] and a study of Chinese middle-aged and elderly men (older than 40 years) [30] estimated 11% (95% CI: 8–14%) and 9% (95% CI: 8–9%) elevated risks of non-accidental mortality, respectively. Two single-center studies [21,25] conducted in China reported similar HRs to our study, but their concentrations of PM_2.5_ were marginally higher than ours. However, all the aforementioned studies relied on satellite-based exposure measurement, which may partially explain the estimation gap between our findings and others’ findings [33]. In this study, we utilized the LUR model to appraise ambient PM_2.5_ based on residential latitude and longitude, which enhanced the ability to identify spatial variability [26,33,34]. The reason why the magnitude of the effect varied from one study to another may be derived from the following aspects. Firstly, the study population varied across different cohorts, and different studies adjusted for different covariates. Secondly, studies have shown that PM_2.5_ concentrations calculated by various assessment methods were different [24,33], causing different magnitudes of estimates. Thirdly, the time window of long-term exposure employed in each study was different [35], which may lead to different estimates of increased mortality for the same increments in PM_2.5_ [22,36,37,38,39]. Fourthly, PM_2.5_ concentrations in developed countries are much lower than those in developing countries [6,22,23,28,35,39]. Lastly, the compositions of PM_2.5_ in different countries are vastly diverse [21,40,41,42,43].

As for cause-specific mortality, a significant association was only observed between long-term exposure to PM_2.5_ and cardiovascular disease mortality. The relationship of PM_2.5_ with cardiovascular disease mortality was relatively stable across different studies. For each 10 μg/m^3^ increment of long-term exposure to PM_2.5_, cardiovascular disease mortality increased, ranging from 6% to 47% in various populations [30,31,32,44]. A meta-analysis including 21 related studies in 2020 found that the HR of the relationship of PM_2.5_ exposure (10 μg/m^3^ per increment) with cardiovascular disease mortality was 1.11 (95% CI: 1.09–1.14) [45]. Nevertheless, extensive epidemiological studies detected significant relationships of long-term exposure to PM_2.5_ with lung cancer, as well as respiratory disease mortality [23,30,32,45,46]. In our study, the mortality rate of respiratory disease and lung cancer might be too insufficient to detect significant associations (due to relatively short follow-up period). Thus, further studies with a longer follow-up period and a larger sample size are warranted in order to improve the study’s power. Furthermore, the relationship between PM_2.5_ and cancer mortality was still controversial [21,39,44,47,48,49,50,51]. One possible reason for this was the interference of non-respiratory cancer, as the occurrence, progression, and deterioration of these cancers might not be directly affected by the atmospheric environment [52].

The underlying mechanisms for the impact of PM_2.5_ exposure on the genesis or death of diseases mainly included inflammation-related cascades, oxidative stress, and DNA damage [1,53,54]. PM_2.5_ can trigger local inflammatory responses and activate cytokines, such as epidermal growth factor and tumor necrosis factor-α, through lymphocytes, endothelial cells, and fibroblasts to cause disease [55]. Furthermore, some components of PM_2.5_ may also induce intracellular oxidative stress, causing excessive reactive oxygen species (ROS), which may result in oxidative DNA damage and eventually cell death [53]. Emissions from motor vehicles contribute significantly to ambient PM2.5 pollution [1] and are known to contain high levels of reactive nitrogen species (RNS) and ROS, which have close ties to carcinogenesis and aging [56,57]. The interaction between ROS and RNS may generate substances with stronger oxidation, thus directly inducing DNA damage [1]. Furthermore, compounds adsorbed by PM_2.5_ may interfere with DNA replication and transcription, which could lead to either cell death or incorrect DNA repair, or even worse, the onset and progression of various cancers [53].

An explicit concentration–response function based on local population facilitates the formulation and revision of air-quality standards and environmental policies [28]. Studies derived from Europe and North America with lower PM_2.5_ exposure (<30 μg/m^3^) found long-term exposure to PM_2.5_ linearly increased mortality [44,58,59]. However, studies covering a wider range of PM_2.5_ concentrations in China detected that this association might be nonlinear, which was mainly embodied in the range of 30–60 μg/m^3^ concentrations [22,24,28,30]. In our study, long-term exposure to PM_2.5_ was linearly associated with non-accidental and cardiovascular disease mortality in the range of 25.57–45.40 μg/m^3^, and we assumed that this concentration range was too narrow to detect potential nonlinear associations. Thus, future cohort studies conducted in countries and areas with a wider range of PM_2.5_ concentrations are warranted to reveal the concentration–response function comprehensively.

In our stratified analysis, people aged ≥65 years had higher cardiovascular disease mortality with PM_2.5_, which was comparable with some studies [23,28,60]. The elderly are susceptible to contracting cardiovascular diseases [61] and tend to be affected by other risk factors. In addition, individuals who took preventive measures on hazy days in our study had a lower risk of PM_2.5_-related respiratory disease mortality, indicating that people ought to take precautions to reduce their exposure to PM_2.5_, such as limiting outings and using air cleaners and masks, especially when air pollution is serious.

The strengths of this study are as follows: Firstly, we utilized the LUR model to estimate PM_2.5_ concentrations according to the latitude and longitude of residential addresses at baseline, combined with meteorological monitoring information and other predictive variables, which was more accurate than other studies that determined the exposure level at the district and county level. In addition, outcome data in this study were obtained from the YHIS, which was precise and allowed us to distinguish the causes of death. However, the limitations of this study should be noted. Above all, this was a single-center study with limited representativeness, and only individuals who participated voluntarily were recruited in our study, resulting in selection bias. As a result, our findings might not be generalizable to the target population. Secondly, we neglected to account for relocating, time spent indoors and outdoors, indoor exposure to PM_2.5_, and commute mode during the follow-up period, which may lead to the misclassification of exposure. Thirdly, we did not adjust for possible confounding effects of different PM_2.5_ components and combined exposure to multiple pollutants. Finally, a longer follow-up period would be desirable since our data were limited to 4.88 years. To further explore the impacts of long-term exposure to PM_2.5_ on human death risks, more representative prospective longitudinal studies with a wider range of exposure concentrations and longer follow-up periods are warranted.

## 5. Conclusions

In summary, this cohort study demonstrated that long-term exposure to PM_2.5_ linearly augmented non-accidental and cardiovascular disease mortality among people aged ≥40 years in Yinzhou, China. Stronger associations were observed among individuals aged ≥65 years old and taking no preventive measures on hazy days. The ambient PM_2.5_ in China is continuously decreasing due to the enactment of some environmental policies and economic transition, and our study could provide new insights into long-term exposure to PM_2.5_-related mortality.

## Figures and Tables

**Table 1:** Baseline characteristics across quartiles of PM_2.5_ in the study population.

	Overall	Quartile 1	Quartile 2	Quartile 3	Quartile 4	*p*
	(N = 29,564)	(N = 7503)	(N = 7450)	(N = 7727)	(N = 6884)	
PM_2.5_, median (IQR), μg/m^3^	36.51 (33.27, 39.49)	32.17 (30.87, 32.78)	35.19 (34.69, 36.38)	38.35 (37.76, 38.60)	41.47 (40.42, 41.80)	<0.001 *
Year of follow-up, median (IQR), years	4.88 (4.14, 5.15)	4.43 (4.16, 5.15)	4.22 (4.07, 4.89)	4.35 (4.07, 5.13)	5.16 (5.06, 5.28)	<0.001 *
Age, median (IQR), years	63.17 (57.04, 69.28)	60.26 (54.00, 67.16)	60.62 (54.20, 67.29)	62.86 (55.82, 68.84)	67.50 (63.42, 72.94)	<0.001 *
Male, n (%)	12,142 (41.1)	3004 (40.0)	2696 (36.2)	3328 (43.1)	3114 (45.2)	<0.001
BMI, median (IQR), kg/m^2^	20.59 (18.10, 23.38)	20.93 (18.17, 23.73)	22.07 (19.34, 24.65)	21.08 (18.43, 23.78)	18.86 (17.05, 20.71)	<0.001 *
Marital status, n (%)						
Married	25,905 (87.6)	6666 (88.8)	6667 (89.5)	6716 (86.9)	5856 (85.1)	<0.001
Others	3659 (12.4)	837 (11.2)	783 (10.5)	1011 (13.1)	1028 (14.9)	
Educational level, n (%)						
Illiterate	9960 (33.7)	2693 (35.9)	1987 (26.7)	2744 (35.5)	2536 (36.8)	<0.001
Literate	19,604 (66.3)	4810 (64.1)	5463 (73.3)	4983 (64.5)	4348 (63.2)	
Occupation, n (%)						
Industrial/agricultural	12,109 (41.0)	2836 (37.8)	2196 (29.5)	3554 (46.0)	3523 (51.2)	<0.001
Household/retired	15,374 (52.0)	4161 (55.5)	4422 (59.4)	3612 (46.7)	3179 (46.2)	
Others	2081 (7.0)	506 (6.7)	832 (11.2)	561 (7.3)	182 (2.6)	
Household income, n (%), (CNY/year)						
<30,000	16,790 (56.8)	4956 (66.1)	4663 (62.6)	4858 (62.9)	2313 (33.6)	<0.001
≥30,000	12,774 (43.2)	2547 (33.9)	2787 (37.4)	2869 (37.1)	4571 (66.4)	
Smoking status, n (%)						
Never	23,537 (79.6)	5868 (78.2)	6085 (81.7)	6049 (78.3)	5535 (80.4)	<0.001
Current/former	6027 (20.4)	1635 (21.8)	1365 (18.3)	1678 (21.7)	1349 (19.6)	
Alcohol drinking, n (%)						
Never	24,256 (82.0)	6148 (81.9)	6293 (84.5)	6292 (81.4)	5523 (80.2)	<0.001
Current/former	5308 (18.0)	1355 (18.1)	1157 (15.5)	1435 (18.6)	1361 (19.8)	
Tea drinking, n (%)						
Current/former	2969 (10.0)	729 (9.7)	672 (9.0)	752 (9.7)	816 (11.9)	<0.001
Never	26,595 (90.0)	6774 (90.3)	6778 (91.0)	6975 (90.3)	6068 (88.1)	
Sleep quality, n (%)						
Good	24,079 (81.4)	6340 (84.5)	6093 (81.8)	6161 (79.7)	5485 (79.7)	<0.001
Bad	5485 (18.6)	1163 (15.5)	1357 (18.2)	1566 (20.3)	1399 (20.3)	
Preventive measures on hazy days, n (%)						
Yes	10,750 (36.4)	1457 (19.4)	2404 (32.3)	2375 (30.7)	4514 (65.6)	<0.001
No	18,814 (63.6)	6046 (80.6)	5046 (67.7)	5352 (69.3)	2370 (34.4)	
History of chronic disease, n (%)						
No	12,936 (43.8)	3775 (50.3)	3241 (43.5)	3348 (43.3)	2572 (37.4)	<0.001
Yes	16,628 (56.2)	3728 (49.7)	4209 (56.5)	4379 (56.7)	4312 (62.6)	

Abbreviations: PM_2.5_: fine particulate matter; IQR: interquartile range; BMI: body mass index; CNY: Chinese Yuan. * Calculated by Kruskal–Wallis test.

**Table 2:** Hazard ratios and 95% confidence intervals (95% CIs) for the associations of PM_2.5_ with risk of non-accidental and cause-specific mortality.

	Death Cases	Hazard Ratio (95% CI)			
		Model 1 ^a^	Model 2 ^b^	Model 3 ^c^	Model 4 ^d^
Non-accidental	1082				
Q1 (≤33.27)	199	1.00 (Ref)	1.00 (Ref)	1.00 (Ref)	1.00 (Ref)
Q2 (>33.27 and ≤36.51)	174	0.97 (0.79–1.19)	1.00 (0.81–1.22)	1.09 (0.89–1.34)	1.09 (0.88–1.34)
Q3 (>36.51 and ≤39.49)	240	1.00 (0.83–1.21)	0.99 (0.82–1.20)	1.04 (0.86–1.25)	1.03 (0.85–1.25)
Q4 (>39.49)	469	1.30 (1.10–1.53)	1.31 (1.10–1.55)	1.26 (1.06–1.51)	1.26 (1.05–1.51)
*p* for trend		<0.001	0.001	0.015	0.016
Per 10 μg/m^3^ increment		1.33 (1.12–1.59)	1.33 (1.11–1.59)	1.25 (1.04–1.50)	1.25 (1.04–1.50)
Cardiovascular disease	409				
Q1 (≤33.27)	69	1.00 (Ref)	1.00 (Ref)	1.00 (Ref)	1.00 (Ref)
Q2 (>33.27 and ≤36.51)	55	0.89 (0.63–1.28)	0.94 (0.66–1.35)	1.06 (0.74–1.51)	1.05 (0.73–1.50)
Q3 (>36.51 and ≤39.49)	104	1.20 (0.88–1.62)	1.16 (0.86–1.58)	1.23 (0.90–1.67)	1.22 (0.90–1.66)
Q4 (>39.49)	181	1.29 (0.98–1.71)	1.32 (0.99–1.76)	1.33 (0.99–1.79)	1.33 (0.99–1.78)
*p* for trend		0.017	0.019	0.038	0.038
Per 10 μg/m^3^ increment		1.40 (1.05–1.86)	1.40 (1.04–1.88)	1.38 (1.02–1.86)	1.38 (1.02–1.86)
Respiratory disease	215				
Q1 (≤33.27)	43	1.00 (Ref)	1.00 (Ref)	1.00 (Ref)	1.00 (Ref)
Q2 (>33.27 and ≤36.51)	36	0.93 (0.60–1.45)	0.99 (0.63–1.55)	1.14 (0.73–1.79)	1.13 (0.72–1.78)
Q3 (>36.51 and ≤39.49)	45	0.89 (0.59–1.36)	0.91 (0.60–1.38)	0.98 (0.64–1.49)	0.97 (0.64–1.49)
Q4 (>39.49)	91	1.25 (0.86–1.81)	1.34 (0.92–1.95)	1.32 (0.89–1.96)	1.32 (0.89–1.95)
*p* for trend		0.182	0.121	0.224	0.228
Per 10 μg/m^3^ increment		1.30 (0.89–1.92)	1.37 (0.92–2.03)	1.31 (0.88–1.96)	1.30 (0.87–1.95)
Cancer	451				
Q1 (≤33.27)	95	1.00 (Ref)	1.00 (Ref)	1.00 (Ref)	1.00 (Ref)
Q2 (>33.27 and ≤36.51)	78	0.91 (0.67–1.23)	0.93 (0.69–1.26)	0.99 (0.73–1.34)	0.99 (0.73–1.34)
Q3 (>36.51 and ≤39.49)	96	0.88 (0.66–1.17)	0.86 (0.65–1.15)	0.89 (0.67–1.18)	0.89 (0.67–1.18)
Q4 (>39.49)	182	1.22 (0.95–1.58)	1.16 (0.90–1.51)	1.14 (0.87–1.50)	1.14 (0.87–1.50)
*p* for trend		0.085	0.247	0.408	0.411
Per 10 μg/m^3^ increment		1.28 (0.98–1.67)	1.20 (0.91–1.57)	1.16 (0.88–1.53)	1.15 (0.87–1.52)
Lung cancer	125				
Q1 (≤33.27)	25	1.00 (Ref)	1.00 (Ref)	1.00 (Ref)	1.00 (Ref)
Q2 (>33.27 and ≤36.51)	19	0.83 (0.46–1.51)	0.90 (0.49–1.64)	0.97 (0.53–1.78)	0.97 (0.53–1.78)
Q3 (>36.51 and ≤39.49)	32	1.12 (0.67–1.90)	1.13 (0.67–1.91)	1.17 (0.69–1.99)	1.17 (0.69–1.99)
Q4 (>39.49)	49	1.34 (0.82–2.20)	1.33 (0.80–2.21)	1.32 (0.78–2.23)	1.32 (0.78–2.23)
*p* for trend		0.134	0.179	0.236	0.237
Per 10 μg/m^3^ increment		1.61 (0.96–2.70)	1.57 (0.93–2.65)	1.53 (0.89–2.61)	1.53 (0.89–2.61)

^a^ Adjusted for age and gender. ^b^ Further adjusted for marital status, educational level, occupation, and household income based on model 1. ^c^ Further adjusted for BMI, smoking status, alcohol consumption, tea consumption, sleep quality, and preventive measures on hazy days based on model 2. ^d^ Further adjusted for history of chronic disease based on model 3.

## Data Availability

The data presented in this study are available on request from the corresponding author. The data are not publicly available due to privacy agreement signed by all participants.

## Supplementary Materials

The following supporting information can be downloaded at: https://www.mdpi.com/article/10.3390/toxics11090727/s1, Methods: Assessment of PM2.5 concentrations; Table S1: Concentration of PM2.5 in death and non-death case; Tabel S2 Akaike information criterion of models with different degrees of freedom; Figure S1: Subgroup analysis for the association of PM2.5 in 10 μg/m^3^ increments with cancer mortality; Figure S2: Subgroup analysis for the association of PM2.5 in 10 μg/m^3^ increments with lung cancer mortality; Figure S3: Subgroup analysis for the association of PM2.5 in 10 μg/m^3^ increments with respiratory disease mortality; Figure S4: Sensitivity analysis for the association of PM2.5 in 10 μg/m^3^ increments and mortality based on the model 4.
