# Supplementary material for: Long-Term Exposure to PM2.5 and Mortality: A Cohort Study in China
Source: Toxics. 2023 Aug 24;11(9):727. doi: 10.3390/toxics11090727 (PMC10534778; doi:10.3390/toxics11090727)
Supplement: Supplementary file 1 [file toxics-11-00727-s001.zip › toxics-2516730-supplementary.pdf]

Supplementary appendix

Long-term exposure to $PM_{2.5}$ and mortality: a cohort study in China

Supplementary methods: Assessment of $PM_{2.5}$ concentrations.

Table S1 Concentration of $PM_{2.5}$ in death and non-death case.

Tabel S2 Akaike information criterion of models with different degrees of freedom.

Figure S1. Subgroup analysis for the association of $PM_{2.5}$ in 10 μg/m³ increments with cancer mortality.

Figure S2. Subgroup analysis for the association of $PM_{2.5}$ in 10 μg/m³ increments with lung cancer mortality.

Figure S3. Subgroup analysis for the association of $PM_{2.5}$ in 10 μg/m³ increments with respiratory disease mortality.

Figure S4. Sensitivity analysis for the association of $PM_{2.5}$ in 10 μg/m³ increments and mortality based on the model 4.

Supplementary methods: Assessment of $PM_{2.5}$ concentrations.

High-spatiotemporal-resolution land-use regression (LUR) models were developed to estimate the monthly outdoor levels of $PM_{2.5}$ based on each participant's baseline address. Briefly, it was composed of 4 main steps as follows:

1. Data sources: Daily air pollution monitoring and meteorological data in Zhejiang Province were obtained from the Chinese Ecology and Environment Ministry with technical support from the Qingyue environmental protection information technology service center (http://data.epmap.org/). Geographic data (distance to nearest road, elevation, types of land cover in different buffers, latitude, longitude, etc.) were derived from Geographic Information System (GIS).
2. Predictors identification: Land use predictors (population density, elevation, distance to nearest road, percentage of land cover in different buffers) and meteorological data (temperature ℃, relative humidity %, and wind speed m/s) were included in the model. These covariates were firstly natural-log transformed, and a constant was added to obtain a uniform distribution before entering the model.
3. Model construction: We utilized generalized additive models (GAMs) to predict monthly outdoor levels of $PM_{2.5}$, which was firstly developed with some spatiotemporal spline variables, including latitude, longitude, year, month, and season. Covariates listed in Step 2 were then added to the model one by one
4. Model evaluation: Akaike Information Criterion (AIC) and Generalized Cross Validation (GCV) scores were used to estimate model performance. For 10-fold cross-validation, we randomly selected 90% of the data as the training set and the rest 10% was held out for validation in 10 separate iterations. Overall, the final $PM_{2.5}$ LUR model performance was reasonably accurate and precise, with 10-fold cross-validation R^2 of 0.75.

Table S1 Concentration of $PM_{2.5}$ in death and non-death case.

	**Non-death case**	**Death case**
**Non-accidental**		
n	28482	1082
$PM_{2.5}$, median (IQR), μg/m³	36.51 (33.27, 39.18)	38.78 (35.19, 41.47)
**Cardiovascular disease**		
n	29155	409
$PM_{2.5}$, median (IQR), μg/m³	36.51 (33.27, 39.49)	38.78 (35.45, 41.47)
**Respiratory disease**		
n	29349	215
$PM_{2.5}$, median (IQR), μg/m³	36.51 (33.27, 39.49)	39.16 (35.19, 41.47)
**Cancer**		
n	29113	451
$PM_{2.5}$, median (IQR), μg/m³	36.51 (33.27, 39.49)	38.52 (34.65, 41.02)
**Lung cancer**		
n	29439	125
$PM_{2.5}$, median (IQR), μg/m³	36.51 (33.27, 39.49)	39.16 (35.19, 41.47)

Tabel S2 Akaike information criterion of models with different degrees of freedom

	**Knots = 3**	**Knots = 4**	**Knots = 5**
**Non-accidental**	15918.01	15918.80	15918.61
**Cardiovascular disease**	5722.03	5723.85	5725.34
**Respiratory disease**	3187.35	3189.22	3190.75
**Cancer**	7046.25	7047.07	7049.23
**Lung cancer**	1981.00	1983.00	1984.92

Characteristics	Subgroup	Death (%)	HR(95% CI)	$P_{interaction}$
All		451 (100)	1.16 (0.87-1.52)	
Age (years)	< 65	123 (27.27)	1.30 (0.77-2.20)	0.696
	≥ 65	328 (72.73)	1.06 (0.76-1.47)	
Sex	Male	297 (65.85)	1.15 (0.82-1.61)	0.508
	Female	154 (34.15)	1.17 (0.71-1.91)	
Educational level	Iliteracy	191 (42.35)	1.12 (0.73-1.70)	0.614
	Literacy	260 (57.65)	1.18 (0.81-1.71)	
Occupation	Industry or agriculture	245 (54.32)	1.21 (0.82-1.77)	0.898
	Other	206 (45.68)	1.08 (0.72-1.61)	
Household income (CNY/year)	≥ 30,000	158 (35.03)	1.07 (0.67-1.71)	0.716
	< 30,000	293 (64.97)	1.19 (0.84-1.69)	
BMI (kg/m²)	18.5-24	230 (51.00)	1.21 (0.81-1.80)	0.575
	< 18.5	188 (41.69)	1.00 (0.66-1.52)	
	≥ 24	33 (7.32)	1.11 (0.34-3.59)	
Smoking status	Never	304 (67.41)	1.22 (0.86-1.72)	0.551
	Current/former	147 (32.59)	1.08 (0.67-1.72)	
Alcohol drinking	Never	324 (71.84)	1.17 (0.84-1.63)	0.992
	Current/former	127 (28.16)	1.13 (0.67-1.89)	
Tea drinking	Current/former	67 (14.86)	1.56 (0.76-3.23)	0.540
	Never	384 (85.14)	1.10 (0.82-1.49)	
Sleep quality	Good	373 (82.71)	1.14 (0.84-1.55)	0.962
	Bad	78 (17.29)	1.16 (0.59-2.28)	
Preventive measures in hazy days	Yes	203 (45.01)	1.13 (0.73-1.73)	0.473
	No	248 (54.99)	1.18 (0.82-1.71)	
History of chronic disease	No	170 (37.69)	1.07 (0.69-1.66)	0.952
	Yes	281 (62.31)	1.22 (0.85-1.74)	

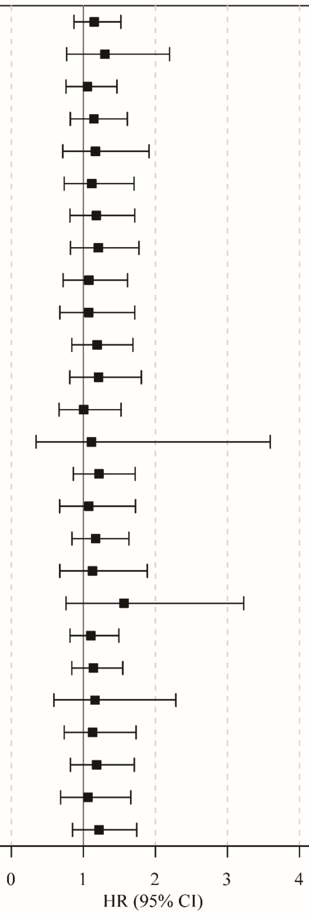

Figure S1. Subgroup analysis for the association of $PM_{2.5}$ in 10 μg/m³ increments with cancer mortality.

Characteristics	Subgroup	Death (%)	HR(95% CI)	$P_{interaction}$
All		125 (100)	1.53 (0.89-2.61)	
Age (years)	< 65	38 (30.40)	1.83 (0.69-4.85)	0.453
	≥ 65	87 (69.60)	1.33 (0.70-2.54)	
Sex	Male	90 (72.00)	1.35 (0.73-2.50)	0.105
	Female	35 (28.00)	2.52 (0.84-7.58)	
Educational level	Iliteracy	58 (46.40)	1.65 (0.76-3.58)	0.523
	Literacy	67 (53.60)	1.39 (0.66-2.89)	
Occupation	Industry or agriculture	76 (60.80)	1.77 (0.88-3.56)	0.929
	Other	49 (39.20)	1.35 (0.59-3.07)	
Household income (CNY/year)	≥ 30,000	42 (33.60)	1.01 (0.42-2.47)	0.745
	< 30,000	83 (66.40)	1.78 (0.91-3.48)	
BMI (kg/m²)	18.5-24	60 (48.00)	2.18 (0.98-4.88)	0.709
	< 18.5	54 (43.20)	1.03 (0.47-2.25)	
	≥ 24	11 (8.80)	0.96 (0.13-6.98)	
Smoking status	Never	68 (54.40)	2.10 (0.98-4.49)	0.288
	Current/former	57 (45.60)	1.08 (0.50-2.33)	
Alcohol drinking	Never	86 (68.80)	1.51 (0.79-2.88)	0.856
	Current/former	39 (31.20)	1.52 (0.59-3.94)	
Tea drinking	Current/former	27 (21.60)	1.94 (0.59-6.33)	0.302
	Never	98 (78.40)	1.41 (0.77-2.57)	
Sleep quality	Good	104 (83.20)	1.33 (0.74-2.38)	0.570
	Bad	21 (16.80)	2.27 (0.56-9.23)	
Preventive measures in hazy days	Yes	55 (44.00)	1.26 (0.56-2.85)	0.146
	No	70 (56.00)	1.88 (0.93-3.79)	
History of chronic disease	No	50 (40.00)	1.39 (0.60-3.25)	0.858
	Yes	75 (60.00)	1.61 (0.80-3.23)	

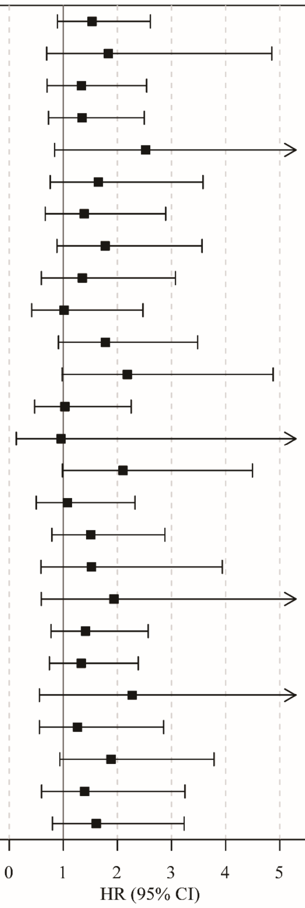

Figure S2. Subgroup analysis for the association of $PM_{2.5}$ in 10 μg/m³ increments with lung cancer mortality.

Characteristics	Subgroup	Death (%)	HR(95% CI)	$P_{interaction}$
All		215 (100)	1.31 (0.87-1.95)	
Age (years)	< 65	47 (21.86)	1.08 (0.46-2.49)	0.437
	≥ 65	168 (78.14)	1.33 (0.84-2.11)	
Sex	Male	155 (72.09)	1.30 (0.81-2.08)	0.256
	Female	60 (27.91)	1.43 (0.65-3.15)	
Educational level	Iliteracy	119 (55.35)	1.48 (0.87-2.54)	0.307
	Literacy	96 (44.65)	1.14 (0.63-2.08)	
Occupation	Industry or agriculture	120 (55.81)	1.31 (0.76-2.25)	0.512
	Other	95 (44.19)	1.45 (0.80-2.63)	
Household income (CNY/year)	≥ 30,000	63 (29.30)	1.28 (0.61-2.68)	0.778
	< 30,000	152 (70.70)	1.28 (0.79-2.06)	
BMI (kg/m²)	18.5-24	87 (40.47)	2.10 (1.07-4.10)	0.500
	< 18.5	115 (53.49)	0.95 (0.56-1.62)	
	≥ 24	13 (6.05)	1.40 (0.22-8.95)	
Smoking status	Never	140 (65.12)	1.29 (0.78-2.13)	0.868
	Current/former	75 (34.88)	1.28 (0.65-2.52)	
Alcohol drinking	Never	162 (75.35)	1.12 (0.71-1.78)	0.203
	Current/former	53 (24.65)	2.19 (0.94-5.07)	
Tea drinking	Current/former	31 (14.42)	2.02 (0.66-6.19)	0.248
	Never	184 (85.58)	1.23 (0.80-1.90)	
Sleep quality	Good	171 (79.53)	1.31 (0.83-2.06)	0.946
	Bad	44 (20.47)	1.17 (0.48-2.83)	
Preventive measures in hazy days	Yes	100 (46.51)	1.02 (0.56-1.83)	**0.040**
	No	115 (53.49)	1.78 (1.03-3.07)	
History of chronic disease	No	76 (35.35)	1.70 (0.86-3.37)	0.239
	Yes	139 (64.65)	1.14 (0.69-1.89)	

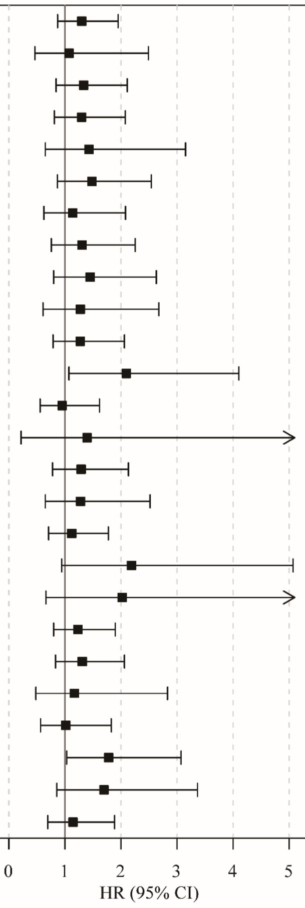

Figure S3. Subgroup analysis for the association of $PM_{2.5}$ in 10 μg/m³ increments with respiratory disease mortality.

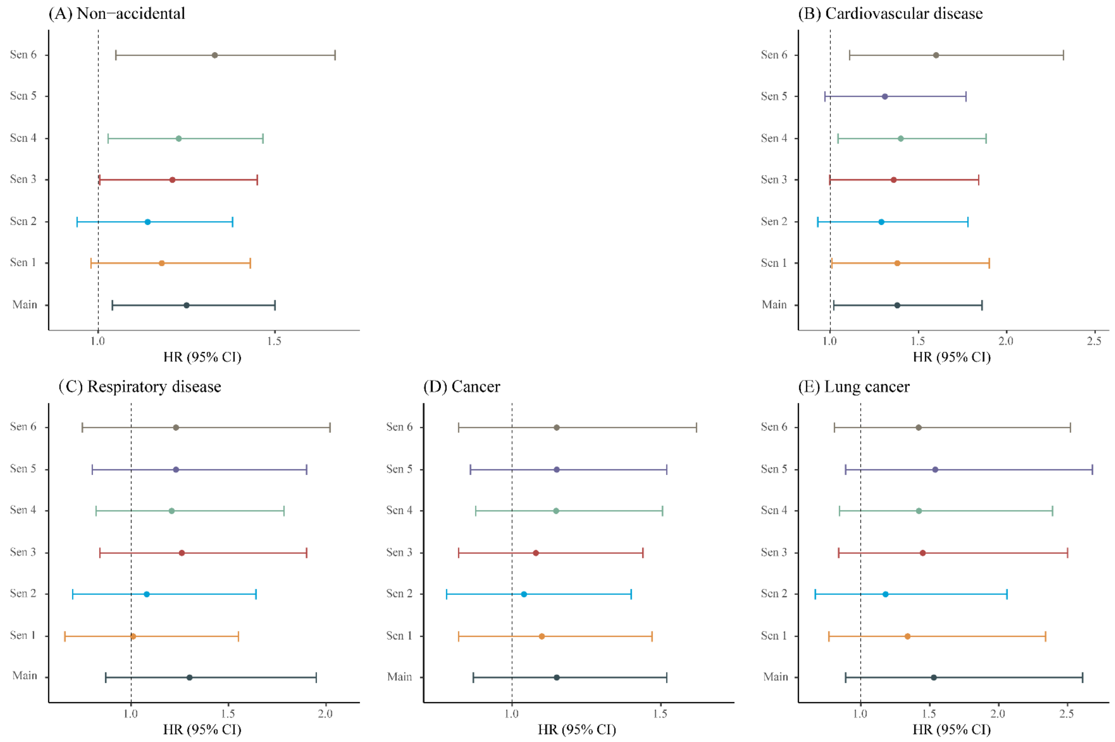

Figure S4. Sensitivity analysis for the association of $PM_{2.5}$ in 10 μg/m³ increments and mortality based on the model 4. Main: main analysis for the association of $PM_{2.5}$ in 10 μg/m³ increment with mortality; Sen 1: excluding participants who died within the first year after enrollment; Sen 2: utilizing the 1-year average $PM_{2.5}$ concentrations prior to the baseline as the exposure; Sen 3: excluding participants with major diseases at baseline; Sen 4: using multiple imputation for missing data; Sen 5: using a competing risk model; Sen 6: using a propensity score weighting method.
